# Genotype-by-Environment Interaction in Red Tilapia (*Oreochromis* spp.): Implications for Genetic Parameters and Trait Performance

**DOI:** 10.3390/genes16080966

**Published:** 2025-08-18

**Authors:** Tran Huu Phuc, Pham Dang Khoa, Nguyen Thi Dang, Tran Thi Mai Huong, Huynh Thi Bich Lien, Vo Thi Hong Tham, Nguyen Huynh Duy, Nguyen Hong Nguyen

**Affiliations:** 1 Research Institute for Aquaculture No.2, 116 Nguyen Dinh Chieu Street, District 1, Ho Chi Minh City 70000, Vietnam; tranhuuphuc30@gmail.com (T.H.P.); pdk19045@gmail.com (P.D.K.); nguyenthidang34@gmail.com (N.T.D.); lien15041993@gmail.com (H.T.B.L.); hongtham.dhntts11@gmail.com (V.T.H.T.); duynh09@gmail.com (N.H.D.); 2 Center for Bioinnovation, University of the Sunshine Coast, Locked Bag 4, Maroochydore DC, QLD 4558, Australia; t_t115@student.usc.edu.au; 3 School of Science, Engineering and Technology, University of the Sunshine Coast, Locked Bag 4, Maroochydore DC, QLD 4558, Australia

**Keywords:** genetic parameters, quality and survival traits, genotype-by-environment interaction

## Abstract

The intensive farming of aquaculture species such as red tilapia (*Oreochromis* spp.) across diverse production systems can lead to changes in genetic parameters and responses of economically important traits in this species. This study represents the first attempt to understand these changes in growth traits (body weight, total length), quality attributes (body colour), and survival rate in red tilapia. Data for these traits were collected from 75,950 individual fish, progeny of 970 full-sib families (comprising 970 dams and 486 sires); they were selected for high body weight and evaluated in two distinct culture environments: fresh- and saltwater ponds. A multi-trait mixed model was employed to estimate genetic parameters and selection responses. Genetic variance estimates for the quality and survival traits varied across the two environments. However, genetic correlations among the traits studied were similar between fresh and saline water. Furthermore, significant G × E interactions, particularly for the quality and survival traits, were evidenced by divergent genetic correlations (*r_g_* = 0.57–0.83) between homologous traits across different environments. The findings emphasise the importance of incorporating G × E interactions into the selection program for red tilapia, particularly when the breeding objectives extend to include quality and survival traits. Selection strategies should consider the prevailing culture system—for instance, favouring genotypes suited to the freshwater pond environment over those adapted to the saltwater environment. Continual assessment of full-sib groups across these environments is recommended to refine our understanding of G × E interactions and optimise future breeding programs for red tilapia. This may involve selecting genotypes capable of consistent performance across environments or developing environment-specific breeding programs.

## 1. Introduction

Intensive farming practices for red tilapia (*Oreochromis* spp.) encompass a spectrum of diverse culture systems, from small-scale freshwater ponds to expansive open-river cages [1]. In major producing countries like those in Asia, prevalent production systems include freshwater ponds and freshwater cages, with the freshwater pond system dominating due to its efficacy [2]. While cage culture was once utilised, its declining trend stems from prohibitive production costs tied to initial investments. Recently, saltwater environments have started increasing, due to the protrusion of seawater in many deltas round the world, such as the Mekong Delta or the Ganges–Brahmaputra of Bangladesh and India [3,4]. Farming of red tilapia in saltwater pens or ponds has increased in the Mekong region. Typical distinctions among the two primary systems (freshwater and saline ponds) span fish density, water depth, temperature, flow rate, feeding regime, and grow-out duration (T.H. Phuc person com.).

The differences in these culture systems employed in red tilapia present challenges regarding potential genotype-by-environment (G × E) interactions on commercially significant traits [5]. G × E interaction arises when genotypes respond disparately to testing environments, driven primarily by scaling and reranking effects [6]. Scaling G × E interaction manifests when variances differ among environments, while reranking G × E interaction is gauged through genetic correlations between analogous traits across environments. When genetic correlations approach unity, the G × E effect is deemed negligible [7]. However, in the presence of G × E interaction, alternative strategies, such as distinct breeding programs for each production system, warrant consideration, contingent upon cost–benefit analyses [8].

To date, investigations into aquaculture species have primarily scrutinised G × E interactions concerning growth traits [9]. Synthesised findings indicate the potential significance of G × E interaction for body traits, particularly when selection environments diverge significantly from production systems, as observed in various species, like salmonids [10,11], tilapias [12], shrimp/prawns [13], and molluscs, such as the Pacific oyster [14]. Conversely, when selection and production environments align closely, the biological significance of G × E interaction for growth-related traits may diminish [15,16].

Despite these insights, scant published literature addresses G × E interaction for quality and survival traits (e.g., body colour and survival rate) in any aquaculture species, including red tilapia [2,17]. Comprehending G × E interaction is imperative for optimising production performance, particularly in environments divergent from the current selection environment, primarily the freshwater pond. Moreover, given the rising consumer demand for high-quality products, the focus on body colour and flesh-related traits has intensified, given that nearly all red tilapia products are marketed as whole fish or ready-to-eat products [18]. Hence, investigating G × E interaction for these economically pivotal traits is essential for broadening and refining the breeding objectives for red tilapia.

The present study aims to evaluate the magnitudes of G × E interaction for four key traits in red tilapia: body weight, total length, body colour, and survival rate. These traits will be assessed across representative full-sib groups tested in two distinct environments: fresh- and saltwater ponds. The findings from this study are expected to assist in the design and refinement of future genetic enhancement programs for this species.

## 2. Materials and Methods

### 2.1. Experimental Animals

The broodstock used to produce the experimental fish in this study originated from the genetic improvement program for red tilapia at the Research Institute for Aquaculture No.2 (RIA2) in Vietnam. Before breeding, female and male breeders (750 and 350 fish, respectively) were raised separately in hapa nets (5 × 10 × 1 m) assembled in an earthen pond with a surface area of 2000 m^2^ and a water depth of 1.2–1.5 m, stocked at a density of 2 fish/m^2^. The breeders were fed industrial pellets with 40% protein, supplemented with vitamins E and C, zinc, and multivitamins, at a rate of 5% of feed mass and a feeding ration of 3–5% of body weight. At mating, female breeders averaged 880 g, and males averaged 1111 g.

Individually tagged females and males were bred in separate hapa nets (3.0 × 2.5 × 1.0 m) also established in earthen ponds, with a mating ratio of one male to three females. Fertilised eggs collected from individual mating pairs were hatched and incubated separately by family. In each generation, around 120 families (100 selected and 20 control) were produced over a period of 50–70 days (Supplementary Figure S1). Briefly, in the selection line, 2–4 males and 6–8 females with the highest estimated breeding values (EBVs) for body weight were selected from each family to produce the next generation. The control group was randomly established based on the mean body weight of the population. Mating between full- and half-sib families was avoided to minimise inbreeding and prevent black spot pigmentation. The same selection and mating procedures were applied across all generations. After hatching, fry from each family were reared separately in replicated hapa nets and fed a high-protein diet with 45% protein content. After approximately 117 days (ranging from 84 to 150 days), fingerlings reached an average weight of 10.5 ± 6.6 g for physical tagging. Each fish was marked using a Passive Integrated Transponder (PIT) tag with clove oil as an anaesthetic, and then conditioned in tanks for 2–3 days before being sent to freshwater and saltwater ponds for performance testing. An average of 50 tagged fish from each family were used in each testing environment.

### 2.2. Testing Environments and Data Recording

In all generations, offspring of both the selection and control groups were evaluated for their performance in two environments: freshwater and saline ponds. In the freshwater environment, fish were raised in a 2000 m^2^ earthen pond with a water depth of 1.5 m at the National Centre for Southern Freshwater Aquaculture Breeding of RIA2 in Tien Giang Province, Vietnam. In the saltwater environment, fish were raised in similarly sized 2000 m^2^ ponds with a water depth of 1.5 m and an average salinity of 10–15‰, located in Long Vinh, Duyen Hai, Tra Vinh Province.

During the grow-out period, fish were fed industrial pellets with 30% protein at 3–5% of their body weight. Probiotics (*Bacillus megaterium, Bacillus polymyxa, Bacillus subtilis, Bacillus licheniformis*, and *Saccharomyces cerevisiae*) were applied periodically to enhance water quality. Water quality parameters were closely monitored to minimise variation between ponds (temperature: 28–30 °C, oxygen > 5 mg/L, and ammonium nitrogen < 1 mg/L). Additionally, the water was changed twice a month, with 30% of the pond volume replaced each time.

After a 223-day growth period, the fish were harvested using seine nets and pond drainage. Data collected included individual fish ID (traced through PIT tags), family information, sex, and four commercial traits: body weight, total length, colour, and survival. Body weight was recorded with an electronic scale (accurate to 0.1 g), and total length was measured with a ruler to 0.5 mm precision. Colour was visually assessed based on the presence of dark spots on the body surface, categorised as ‘pass’ (no or few dark spots covering <5% of the body) or ‘not pass’ (many dark spots).

### 2.3. Statistical Analysis

#### 2.3.1. Heritability and Correlations

Variance components including additive genetic ($\sigma_{A}^{2}$), common full-sib ($\sigma_{c}^{2}$), environmental ($\sigma_{E}^{2}$), and phenotypic ($\sigma_{P}^{2}$) variances were estimated using Echidna software 1.2 (Gilmour et al., 2022) [19], which employs a Restricted Maximum Likelihood (REML)-Average Information algorithm and sparse matrix methods for fitting the mixed model. The mixed model accounts for possible systematic fixed and random effects to enable the separation of different variance components, as described below.

For the growth traits (weight and length), the linear mixed model was used, as described in Equation (1):

(1) *y_ijklmno_* = *μ* + *G_i_* + *L_j_* + *S_k_* + *Age_l_* + *a_m_* + *c_n_* + *e_ijkmno_*

where *y_ijklmno_* = the trait observations; *μ* is the population mean; *G_i_ = generation* is the class fixed effect, with *i* = 1 to 8; *L_j_* is the fixed effect of line (selection or control); *S_k_* is the fixed effect of sex, with 2 levels (male or female); *Age_l_* is a linear covariate of age from birth to harvest; *a_m_* is the random additive genetic effect of individual fish in the pedigree; *c_n_* represents the maternal and common full-sib effects due to the separate rearing period of each family from birth to tagging; and *e_ijklmno_* represents the error residuals. Fixed effects were determined using a general linear model (GLM), and random terms were assessed using log-likelihood ratio tests (*p* < 0.05). Preliminary analyses included the Kolmogorov–Smirnov test for normality and Levene’s and F-tests for variance homogeneity. Post hoc comparisons of means used Tukey’s test at a 5% significance level.

The heritability (*h^2^*) and common full-sib effects (*c^2^*) were estimated as follows:

$$h^{2}=\frac{\sigma_{A}^{2}}{\sigma_{A}^{2}+\sigma_{C}^{2}+\sigma_{E}^{2}}$$

$$c^{2}=\frac{\sigma_{C}^{2}}{\sigma_{A}^{2}+\sigma_{C}^{2}+\sigma_{E}^{2}}$$

Regarding colour and survival (binary traits), in addition to model 1, the generalised linear mixed model with a logit function (model 2) was used to calculate the variance components. The same fixed effects as model 1 were included, but the random terms fitted were the genetic effects of sires and dams, along with the common full-sib effects.

(2) $$\log(\frac{p_{ijklmnop}}{1-p_{ijklmnop}})=\mu +G_{i}+L_{j}+S_{k}+{\mathrm{Age}}_{l}+s_{m}+d_{n}+f_{p}+e_{\mathrm{ijkmno}}$$

where *G_i_*, *L_j_*, *S_k_*, and *Age_l_* are the fixed effects, as defined above, and *s_m_* and *d_n_* are the random effects of sires and dams in the model, respectively, while the common full-sib effects are indicated by *f_p_*.

$$h^{2}=\frac{4\sigma_{s}^{2}}{\sigma_{s}^{2}+\sigma_{d}^{2}+\sigma_{e}^{2}\frac{\pi^{2}}{3}}$$

$$c^{2}=\frac{\sigma_{f}^{2}}{\sigma_{s}^{2}+\sigma_{f}^{2}+\sigma_{e}^{2}\frac{\pi^{2}}{3}}$$

where $\sigma_{a}^{2}$ is the additive genetic variance ( $\sigma_{a}^{2}=4\times \sigma_{s}^{2}$), $\sigma_{s}^{2}$ is the sire variance, $\sigma_{d}^{2}$ is the dam variance, $\sigma_{f}^{2}$ is the common full-sib variance, and $\sigma_{e}^{2}=1$.

Phenotypic (*r_p_*) and genetic (*r_g_*) correlations between traits were estimated using a series of bivariate models, as described in Equation (1).

$$r_{p/g}=\frac{\sigma_{12}}{\sqrt{\sigma_{1}^{2}}\times \sqrt{\sigma_{2}^{2}}}$$

where $\sigma_{12}$ is the covariance between pairs of traits, and $\sigma_{1}^{2}$ and $\sigma_{2}^{2}$ are the phenotypic and genetic variance in individual traits, respectively.

#### 2.3.2. Genotype-by-Environment Interaction

To evaluate genotype-by-environment (G × E) interactions, an initial analysis using a fixed-effects model that included the G × E term as a factor revealed a significant G × E interaction effect (*p* < 0.05). The study then applied a multivariate model, treating phenotypic expressions in freshwater and saltwater ponds as separate traits, thereby assuming no phenotypic covariation between corresponding traits across environments. The genetic correlation between these homologous trait expressions in freshwater and saltwater ponds was estimated as $r=\frac{\sigma_{FS}}{\sqrt{\sigma_{F}^{2}}\sqrt{\sigma_{S}^{2}}}$, where $\sigma_{FS}$ is the estimated additive genetic covariance of homologous traits (weight, length, colour, and survival) between freshwater (F) and saline (S) ponds, and $\sigma_{F}^{2}$ and $\sigma_{S}^{2}$ are the additive genetic variances of analogous traits in freshwater and saline ponds, respectively. The multivariate G × E model analysis employed Equation (1) as described above.

#### 2.3.3. Estimation of Genetic Gains

The genetic gains for weight, length, colour, and survival traits were assessed using two distinct methods. The first approach involved calculating gains as the difference in phenotypic least-squares means (LSMs) between the selection line and the control group. In the second approach, gains were measured by comparing the estimated breeding values (EBVs) between the selection and control groups within the same generation.

Each method expressed genetic traits’ responses in actual units, phenotypic standard deviation units (*σ_p_*), genetic standard deviation units (*σ_a_*), and as a percentage of the population mean. The statistical model applied for estimating EBVs for body weight and other traits mirrored the one used to estimate genetic parameters across the population. All analyses were performed using Echidna software, version 1.2 [19].

## 3. Results

### 3.1. Pedigree and Data Structure

Table 1 presents the numbers of dams and sires and their progeny for each culture environment (freshwater and saline water). In each generation, representatives from all families were tested in both environments (3831–6826 individuals in freshwater and 3808–4426 fish in saline water, respectively). Due to limited resources in 2019 and 2020, performance testing was not conducted in saline water during these years. In total, 862 and 108 families from the selection line and control group, respectively, were included in this study (Table 1).

### 3.2. Descriptive Statistics

Fish tested in freshwater ponds reached a significantly higher average weight than those raised in saline water (F-value = 486.2, *p* < 0.01). However, the individual survival rates were slightly lower in freshwater ponds compared to saltwater ponds. The coefficient of variation (CV) for harvest weight was generally similar across both environments. For colour traits, the proportion of fish with dark spots was lower in freshwater than in saline water (Table 2).

### 3.3. Variance Component, Heritability, and Common Full-Sib Effects

Heritability estimates for body traits (weight and length) were higher in freshwater than in saline ponds, due to greater genetic variances (3385.3 vs. 2432.8) in the former vs. the latter environment (Table 3). Similarly, higher heritability for survival in freshwater than in saline water was also observed (0.0275 vs. 0.0054). However, heritability for colour traits showed little difference between the two environments. Both survival and colour traits were further analysed using the LMM model 1, which yielded lower heritability estimates than the GLMM (Table 3). After transforming heritability on the underlying liability (logit) scale to observed values (0/1), the estimates between the two models aligned closely and were consistent across freshwater and saline environments. Variance components attributed to maternal and common full-sib family effects explained 6–21% of the total phenotypic variance, whereas c^2^ effects contributed only 3–6% for survival and colour traits. For example, the c^2^ variances for survival and colour were estimated at 0.0062 and 0.0086, respectively. Collectively, our results suggest that there are heritable genetic components for the four traits studied, enabling genetic improvement through a selection program for this species.

### 3.4. Correlations

Table 4 presents the phenotypic and genetic correlations among the traits studied in fresh and saline water. The genetic correlations between body weight and length were moderately positive, and the magnitudes of their estimates were similar between the two environments studied. Both traits showed non-significant genetic correlations with survival, with estimates being negative in freshwater and positive in saline water. This discrepancy likely arose from higher mortality rates in saline water, where small fish tended to die. Additionally, there were no significant genetic correlations between body traits and colour in both fresh and saline water. Interestingly, survival was negatively correlated with colour, suggesting that fish with dark spots had lower survival rates than their red-coloured counterparts in this population. Overall, the phenotypic correlations had similar signs and magnitudes to the genetic correlations. These findings suggest that there are genetic associations between or among the traits studied, enabling multi-trait selection in genetic enhancement programs for red tilapia.

### 3.5. Genotype-by-Environment Interaction (G × E)

Genetic correlations between homologous trait expressions (weight, length, survival, and colour) in fresh and saline water were estimated to assess G × E interactions (Table 5). For body weight, the genetic correlation between environments was moderate (0.70 ± 0.07) and statistically significant. In contrast, the estimate for total length was low (0.17 ± 0.09). The genetic correlation for survival across environments was not significantly different from zero. Analyses for colour using the full statistical model 1 were also performed, but the log-likelihood failed to converge. Our results suggest that the G × E interaction effects on growth, colour, and survival might hold biological importance in this population of red tilapia.

### 3.6. Direct and Correlated Genetic Responses

Genetic responses to selection aligned well with target traits in desired directions across both environments (Table 6). Significant gains in body weight were achieved in both fresh and saline water. For example, in freshwater, the offspring of this population are expected to weigh, on average, 200.2 g more than the population mean, assuming that the environmental conditions are constant. The gains in body weight across generations for each testing environment (*p* < 0.05) are presented in Supplementary Figure S2. Selection for increased body weight led to positive genetic shifts in total length, survival, and colour. Correlated gains in total length and survival rate were observed, alongside a reduction in the proportion of fish with black spots. Although the survival rate improvements were modest, they were consistent across environments, while the reduction in black spots was more pronounced in freshwater than in saltwater ponds (3.52% vs. 0.17%). Overall, the selection program brought about favourable genetic changes across all four traits.

## 4. Discussion

The current study explored the interplay of genotype–environment (G × E) interactions concerning growth, quality (colour), and survival traits of a red tilapia population undergoing multiple generations of selection. Prevailingly, research in aquaculture has predominantly emphasised body weight, leaving a knowledge gap in understanding G × E interactions for fitness traits like survival and skin colour [20]. Our study demonstrated the G × E interaction effects specific to quality and survival traits in red tilapia. The findings offer valuable insights for refining existing breeding programs tailored to this species. Specifically, our results underscore a significant G × E interaction effect, particularly evident in quality and survival traits, with genetic correlations between trait expressions across the two different environments ranging from −0.17 to 0.70. Drawing parallels from research on other finfish species, such as rainbow trout, we observed similar trends in G × E interactions affecting traits like flesh quality content and muscle density [21]. This highlights the broader relevance of G × E interactions in shaping body colour across finfish species.

Furthermore, our study unveils nuanced differences in heritability estimates across two different environments, indicating the need for tailored breeding and management strategies to reduce heterogeneities in genetic and environmental variances of these traits. In addition to the moderate heritability of growth traits, which remains consistent across the two environments, colour and survival traits exhibit varying heritability estimates, suggesting potential avenues for selective breeding. Genetic correlations among traits also did not differ between the two environments. In both systems, growth traits (weight and length) showed unfavourable genetic correlations with body colour, while the genetic correlation estimates between growth traits and survival were not significant. In other aquaculture species, flesh or body colour exhibited a moderate-to-high positive genetic correlation with body weight in Atlantic salmon [22] and banana shrimp [23]. However, the literature reports on the genetic correlations between growth traits and survival rate are not conclusive, with estimates being positive [24,25,26], negative [27], or not significant [28].

Crucially, we also investigated correlated genetic changes in each environment. Selection for increased body weight in the freshwater pond produced concomitant changes in total length, body colour, and survival rate in both testing environments. The magnitudes of EBV changes in these traits (except body weight) were similar in saline water as in the selection environment of the freshwater pond. In an independent study, Nguyen et al. (2015) [2] reported a reduced response in growth traits (weight, length, width, and depth) in a freshwater cage relative to the selection system in the freshwater pond. Regarding the correlated changes in body colour due to selection for high growth, only [17] reported improvements in the body colour of red tilapia, which is in accordance with the results of our present investigation. Studies in other aquaculture species are lacking for comparison with our findings. Overall, the direction of correlated genetic changes in growth, body colour, and survival rate was consistent with the genetic correlations among these traits, confirming the realised responses achieved in this population of red tilapia.

Due to the interaction effects of genotype by environment (G × E) on the realisation of genetic gains in saltwater ponds, there is a need to review the selection environment for this species. Currently, the culture system for red tilapia (e.g., in Thailand and Vietnam) predominantly relies on freshwater ponds or cages, which account for about 80% of total production. Only 20% of the current production comes from other systems (T.H. Phuc, pers. com.). Given the growing trend of saltwater pond culture systems for red tilapia production, the need to reassess selection environments becomes evident. Alternatively, performance testing of genotypes should be conducted in both freshwater and saltwater ponds each generation to select families showing consistent performance across these environments [29]. Another strategy is to consider running parallel breeding programs, one for each environment, i.e., separately for fresh and saline waters [30]. However, this strategy requires a significant investment that is hardly justified due to our limited resources. We should weigh the relative importance of culture environments in selection indices or consider having an open nucleus to select for superior animals across the studied environments in future breeding programs for red tilapia. With the open nucleus option, there is a risk of bringing new pathogens into the closed selected populations, which may increase the prevalence of disease outbreaks.

Regardless of the strategies employed, careful consideration of economic viability and market dynamics is necessary. Currently, the economic value of red tilapia primarily hinges on harvest weight and body colour. Hence, any shift towards prioritising other traits and quality attributes in breeding programs must align with market demands and economic feasibility. To justify the choice of testing environments in future breeding programs for this species, we performed a rough economic assessment using the same approach as described in our previous study [31] and showed that the economic loss (USD 30,000–50,000/year) due to the G × E interaction for the growth trait is less than the cost of running a new breeding program (USD 300,000/year). Based on both genetic and economic analyses, refining the selection environment seems to be most suitable when the breeding objectives for red tilapia are broadened by including functional traits, and when the fish is priced based on flesh quality attributes (e.g., fillet fat content or chemical composition).

In summary, while our findings substantiate the presence of G × E interactions for quality and survival traits in red tilapia, further research is imperative to validate these observations across diverse environments and populations. Future studies leveraging extensive pedigree data, along with performance testing in other environments, will be instrumental in refining genetic correlation estimates and elucidating the broader implications of G × E interactions in aquaculture.

## 5. Concluding Remarks

Growth traits (weight and length), body colour, and survival appeared to be under similar quantitative genetic control across the tested culture environments (freshwater and saltwater ponds). Minor differences in heritability for the studied traits were due to variations in genetic and environmental variances between the two environments. Genetic correlations between the traits exhibited similar patterns and magnitudes. However, in the two environments under consideration, the implications of G × E interaction on body length and survival traits appear to hold significant biological relevance. Consequently, the current selection program implemented in the freshwater pond may not fully capture the entire spectrum of genetic expression in production systems. Thus, further investigations are warranted to optimise future breeding programs for red tilapia. Overall, our study has shown moderate heritability and favourable genetic correlations among the studied traits across the two examined environments, shedding light on the quantitative genetic basis of novel traits, such as body colour and survival rate, within this emerging aquaculture species. Understanding changes in genetic variations and responses of these phenotypic traits also merits further studies when more data are accumulated in this red tilapia population.

## Figures and Tables

**Table 1:** Number of sires, dams, and progeny in pond and cage environments.

Environment	Generation	Birth Year	Line	Sire	Dam	Family	Progeny
Freshwater	0	2016	Base				16,929
	1	2017	S	99	150	150	3763
			C	8	11	11	201
	2	2018	S	87	223	223	6143
			C	8	18	18	683
	3	2019	S	49	109	109	4952
			C	8	19	19	857
	4	2020	S	47	100	100	4754
			C	8	18	18	796
	5	2021	S	51	100	100	3739
			C	7	16	16	692
	6	2022	S	52	82	82	3860
			C	8	10	10	513
	7	2023	S	49	98	98	5084
			C	0	16	16	806
	**Subtotal**			**486**	**970**	**970**	**36,843**
Saline water	0 (base)	2016	Base	n.a.	n.a.	n.a.	n.a.
	1	2017	S	99	150	150	3519
			C	14	11	11	312
	2	2018	S	87	223	223	4281
			C	8	18	18	145
	3	2019	S	49	109	109	n.a.
			C	8	19	19	n.a.
	4	2020	S	47	100	100	n.a.
			C	8	18	18	n.a.
	5	2021	S	51	100	100	3337
			C	7	16	16	471
	6	2022	S	52	82	82	3815
			C	8	10	10	513
	7	2023	S	49	98	98	4961
			C	0	16	16	824
	**Subtotal**			**486**	**970**	**970**	**22,178**
**Total**				**486**	**970**	**970**	**59,021**

Note: n.a = not available due to limited resources in these years. Base = base or foundation population, S = selection, and C = control. Number of full-sib families corresponds to the number of dams.

**Table 2:** Number of observations (*n*), mean, standard deviation (SD), and coefficient of variation (CV) for traits studied in fresh- and saltwater environments.

Environment	Trait	Unit	n	Mean	SD	CV (%)
Freshwater	Weight	g	22,812	470.8	196.3	41.7
	Length	cm	22,796	22.2	3.1	14.0
	Survival	%	36,843	61.9	48.6	78.5
	Colour	%	22,812	27.2	44.5	163.5
Saltwater	Weight	g	13,843	436.3	181.5	41.6
	Length	cm	13,843	21.7	4.6	21.1
	Survival	%	22,178	62.4	48.4	77.6
	Colour	%	13,843	38.2	48.6	127.3

**Table 3:** Variance components, heritability (h^2^), and common full-sib effects (c^2^) for the four traits recorded in fresh- and saltwater environments.

Environment	Trait	V_A_	V_C_	V_E_	h^2^	c^2^
Freshwater	Weight	3385.3	2743.9	6750.8	0.26 ± 0.025	0.21 ± 0.012
	Length	1.46	0.76	2.03	0.34 ± 0.030	0.18 ± 0.011
	Survival	2.75 × 10^−2^	9.77 × 10^−4^	9.08 × 10^−3^	0.16 ± 0.005	0.04 ± 0.004
	Colour	0.056	0.0086	0.116	0.36 ± 0.050	0.06 ± 0.005
Saltwater	Weight	2432.8	2000	7309.1	0.21 ± 0.044	0.17 ± 0.018
	Length	0.52	0.82	12.9	0.04 ± 0.016	0.06 ± 0.008
	Survival	0.0054	0.0004	0.0124	0.35 ± 0.050	0.03 ± 0.003
	Colour	0.0267	0.0062	0.0851	0.23 ± 0.040	0.05 ± 0.012

V_A_ = genetic variance, V_C_ = common full-sib variance, and V_E_ = environmental variance. Both the heritability (h^2^) and c^2^ estimates are statistically significant (*p* < 0.05).

**Table 4:** Phenotypic (above) and genetic (below) correlations (±S.E.) among traits in fresh- and saltwater environments.

Environment	Trait	Weight	Length	Survival	Colour
Freshwater	Weight		0.557 ± 0.01	−0.002 ± 0.009	−0.004 ± 0.009 ^ns^
	Length	0.586 ± 0.013		−0.013 ± 0.010	0.003 ± 0.009 ^ns^
	Survival	−0.022 ± 0.024 ^ns^	−0.033 ± 0.024 ^ns^		−0.155 ± 0.010
	Colour	0.002 ± 0.028 ^ns^	0.013 ± 0.028 ^ns^	−0.164 ± 0.025	
Saltwater	Weight		0.435 ± 0.010	−0.098 ± 0.015	−0.035 ± 0.014 ^ns^
	Length	0.768 ± 0.032		n/c	−0.124 ± 0.015
	Survival	0.077 ± 0.045	−0.125 ± 0.044		−0.013 ± 0.009 ^ns^
	Colour	−0.159 ± 0.051	−0.155 ± 0.050	n/c	

Note: n/c = the model was not converged. The phenotypic and genetic correlation estimates are significant (*p* < 0.05), except for those with the ‘ns’ superscript; ns = non-significant.

**Table 5:** Genetic correlations (±S.E.) for homologous traits between environments.

Trait	Animal Model	Animal + Full-Sib Group
Weight	0.55 ± 0.04	0.70 ± 0.08
Length	0.57 ± 0.06	0.17 ± 0.09
Survival	−0.17 ± 0.07	0.05 ± 0.03
Colour	0.22 ± 0.06	n/c

Sire and dam model; n/c = the model was not converged.

**Table 6:** Average genetic responses across seven generations of selection, measured as estimated breeding values in actual units (g) and in genetic standard deviation units (*σ_A_*) in fresh- and saltwater environments.

Environment	Trait	LSMs	EBV in Actual Units	*σ_A_*	Percentage of the Mean ^Δ^
Freshwater	Weight (g)	63.5	200.2	2.45	43.7
	Length (cm)	1.3	1.37	0.89	6.24
	Survival (%)	7.1	0.03	0.02	0.04
	Colour (%)	1.5	−1.1	−0.03	−3.52
Saltwater	Weight (g)	25.3	178.4	1.87	38.9
	Length (cm)	0.8	0.92	0.54	4.20
	Survival (%)	−5.3	0.01	0.05	0.14
	Colour (%)	−6.2	−0.03	−0.001	−0.12

LSMs (least-squares means): Differences in LSMs between the selection line and control group. EBV (estimated breeding value): Differences in EBV between the selection line and control group. The observed differences in both LSMs and EBVs were statistically significant (*p* < 0.05). ^Δ^ = (the observed differences in LSMs divided by the population mean) × 100.

## Data Availability

The original contributions presented in this study are included in the article/Supplementary Materials. Further inquiries can be directed to the corresponding author.

## Supplementary Materials

The following supporting information can be downloaded at: https://www.mdpi.com/article/10.3390/genes16080966/s1, Figure S1: The base (foundation) population of red tilapia (Oreochromis spp.) was established in 2016, from which both the selection and control lines originated. Since then, two separate lines or groups have been maintained and selected to produce the next generation, for example, selected parents from each line in 2017 produced offspring for 2018, and so on. In each generation, offspring from both the selection and control groups were performance-tested in both freshwater and saline-water environments. After a grow-out period of approximately 230 days, data were recorded for genetic evaluation and selection for high body weight. This process was repeated over multiple generations (2017–2023); Figure S2: Genetic improvement in body weight expressed in units of genetic standard deviation in both the selection environment (freshwater) and the production system (saline water). Performance testing was not conducted in the saline-water environment during Generations 3 and 4. Generations 1–7 correspond to the years 2017–2023. Dotted lines indicate the linear genetic trend.
