# Supplementary material for: Genotype-by-Environment Interaction in Red Tilapia (Oreochromis spp.): Implications for Genetic Parameters and Trait Performance
Source: Genes (Basel). 2025 Aug 18;16(8):966. doi: 10.3390/genes16080966 (PMC12385727; doi:10.3390/genes16080966)
Supplement: Supplementary file 1 [file genes-16-00966-s001.zip › genes-3790643-supplementary.pdf]

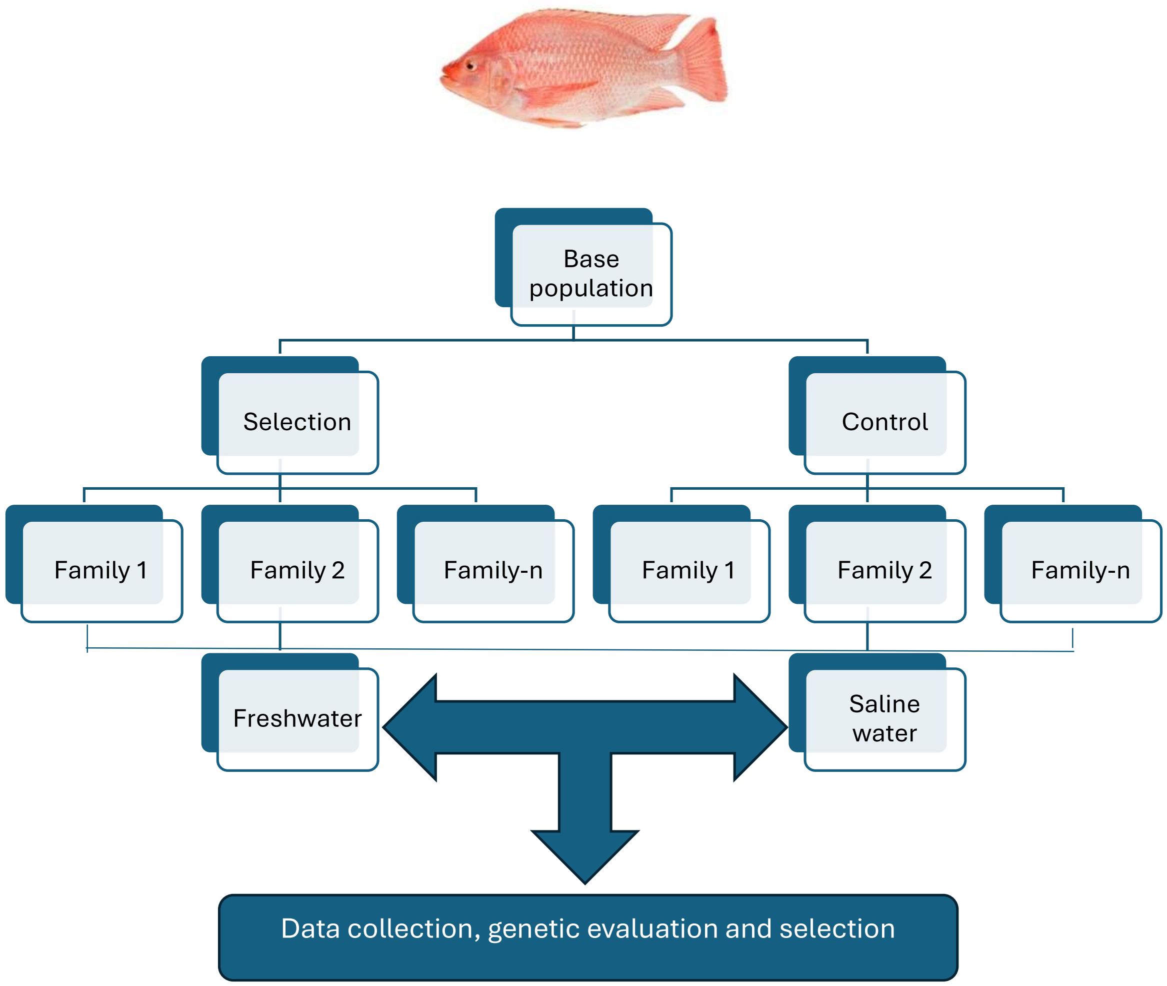

Supplementary Figure S1: The base (foundation) population of red tilapia (Oreochromis spp.) was established in 2016, from which both the selection and control lines originated. Since then, two separate lines or groups have been maintained and selected to produce the next generation, for example, selected parents from each line in 2017 produced offspring for 2018, and so on. In each generation, offspring from both the selection and control groups were performance-tested in both freshwater and saline-water environments. After a grow-out period of approximately 223 days, data were recorded for genetic evaluation and selection for high body weight. This process was repeated over multiple generations (2017–2023).

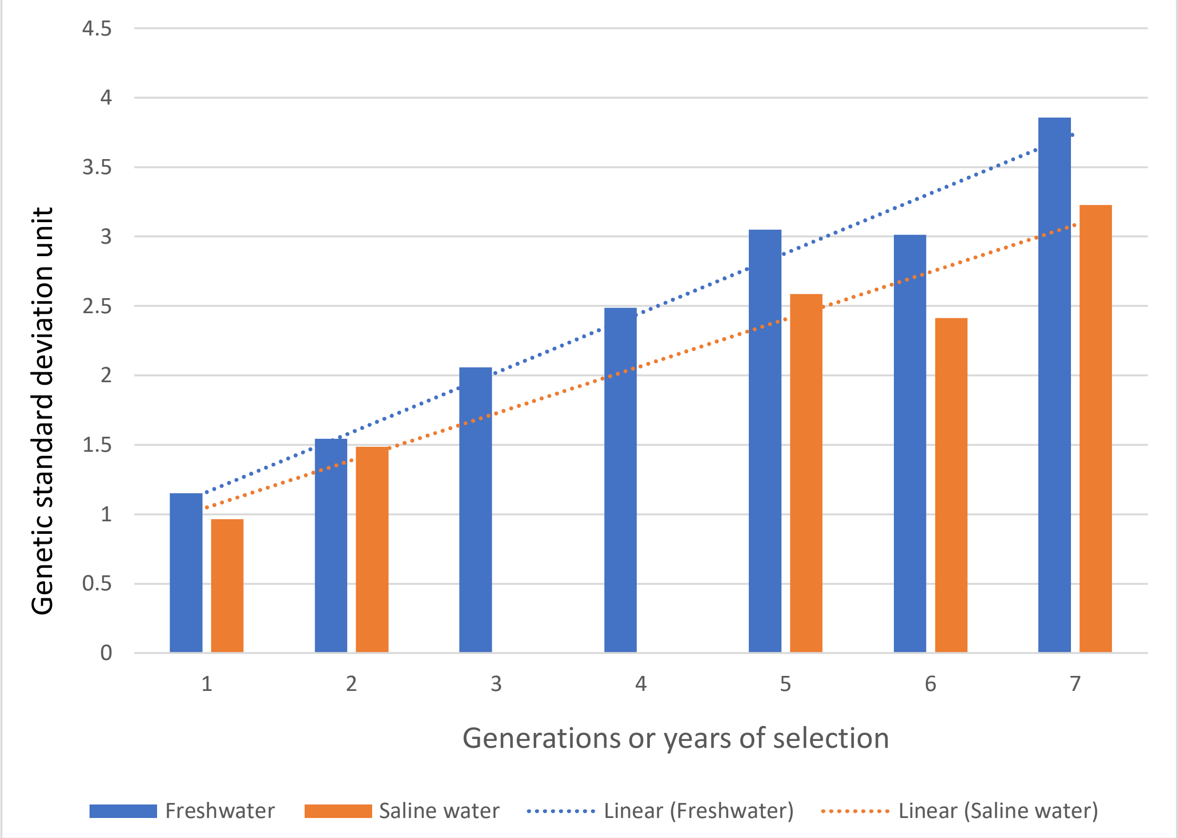

Supplementary Figure S2: Genetic improvement in body weight expressed in units of genetic standard deviation in both the selection environment (freshwater) and the production system (saline water). Performance testing was not conducted in the saline-water environment during Generations 3 and 4. Generations 1–7 correspond to the years 2017–2023. Dotted lines indicate the linear genetic trend, which is statistically significant between the selection and control groups ($P < 0.05$)
